# Low Survival Rates for Pediatric Patients with Tumor Thrombus in Sarcoma

**DOI:** 10.3390/jcm15051806

**Published:** 2026-02-27

**Authors:** Michael J. Colello, Annika Y. Myers, Abigail N. Padilla, Adrian Lin, Brandon Gettleman, Bruce Pawel, Alexander B. Christ

**Affiliations:** 1 Children’s Hospital Los Angeles, Los Angeles, CA 90027, USA; 2 David Geffen School of Medicine, Los Angeles, CA 90095, USA; 3 Department of Orthopedic Surgery, University of California Los Angeles, Los Angeles, CA 90095, USA

**Keywords:** tumor thrombus, sarcoma, survival, osteosarcoma

## Abstract

**Background/Objectives**: Tumor thrombus is an uncommon but serious finding in sarcoma, with limited pediatric data. While adult cases indicate a median survival of ~14 months, outcomes in children remain poorly understood. **Methods**: A retrospective review (1990–2025) was conducted at a single pediatric tertiary center. Patients <18 years with pathologically confirmed bone or soft tissue sarcoma and radiographic or histologic evidence of tumor thrombus were included. Minimum follow-up was 3 years or until end of life. The primary outcome was survival after tumor thrombus diagnosis. **Results**: Thirteen patients (nine males, four females) met the inclusion criteria. The median age at sarcoma diagnosis was 10.5 years. Osteosarcoma was the most common subtype (69.2%), with 76.9% of tumors arising in bone. Disease was localized in 53.8% and metastatic in 46.2% at presentation. Tumor thrombus was contiguous in 61.5% and noncontiguous in 38.5%. The median time from sarcoma diagnosis to death was 44.2 months; from tumor thrombus diagnosis to death, this was 15.2 months. The overall survival after tumor thrombus diagnosis was 30.8%. **Conclusions**: Pediatric sarcoma with tumor thrombus is associated with poor prognosis, and surgical intervention did not appear to result in long-term survival in this small series. Tumor thrombus may be noncontiguous from the primary tumor, emphasizing the importance of advanced imaging and its implications for treatment planning and counseling.

## 1. Introduction

Sarcomas are malignant tumors that make up over 20% of all pediatric solid cancers and can be divided into two main groups: soft tissue sarcomas and bone sarcomas [1,2,3]. They are diverse, with more than 60 different subtypes [1,3]. This diversity leads to a variety of clinical phenotypes, with soft tissue sarcomas usually presenting as a deep, painless mass, and bone sarcomas often causing deep bone pain, swelling, or stiffness [1,3]. The overall five-year survival rate ranges from 60% to 75%, depending on the specific type of sarcoma [4,5,6,7].

Tumor thrombus is a rare event where an organized collection of tumor cells invades an adjacent blood vessel. It is most commonly observed in carcinomas such as renal cell carcinoma or hepatocellular carcinoma [8,9]. Tumor thrombus can significantly impact cancer staging, prognosis, and treatment, and may lead to life-threatening complications including venous thromboembolism, major bleeding, or cardiac dysfunction [9]. The presence of tumor thrombus is often a poor prognostic indicator, with mortality rates ranging from 34% to 50% depending on the type of malignancy [9,10,11,12].

Intra-vascular invasion of the tumor not only affects mortality, but also complicates surgical management [8]. Treatment of sarcoma includes radiation therapy, chemotherapy and local control surgery, depending on the histology [13,14]. The cornerstone of local control surgery is achieving a negative margin resection. It has been well defined that positive margin resections in sarcoma are associated with increased rates of local recurrence, decreased survival, and the need for additional adjuvant therapies or morbid surgeries [15,16,17,18]. In order to achieve a negative margins in the presence of adjacent tumor thrombus, the involved vasculature must either be resected or a thrombectomy performed along with the resection of the primary tumor. When major vascular structures are involved, this often necessitates vascular reconstruction in order to preserve the limb which introduces significant morbidity and additional complications to the surgery [19,20]. Thus, since the presence of tumor thrombus in sarcoma indicates a poor prognosis and the surgical treatment carries a high morbidity, patient-surgeon discussion of operative versus nonoperative measures is critical.

The incidence with which tumor thrombus is associated with sarcomas is not well-defined in the literature, with most reports consisting of case studies [21,22,23,24,25]. The specific incidence of tumor thrombus non-contiguous to the primary tumor is also unclear. Navalkele et al. reported a single case of a proximal humerus osteosarcoma with a non-contiguous tumor thrombus extending into the heart [21], Atagi et al. reported a single case of a retroperitoneal Ewing’s sarcoma with tumor thrombus in the inferior vena cava extending into the right atrium [23], and Nakata et al. described a single case of a sacral osteosarcoma with contiguous tumor thrombus in the inferior vena cava associated with an IVC filter [25]. However, tumor thrombus has been shown to be common in pelvic sarcomas, with rates around 10% [26], and as high as 51% in osteosarcomas of the pelvis [12].

Research on patient survival with tumor thrombus in sarcoma is scarce and mainly focused on adults with pelvic sarcomas. Yedururi et al. found 20 patients with pelvic osteosarcoma and tumor thrombus, with a survival rate of 25% at 5 years [12]. Notably, only half of these patients underwent surgical resection of the primary tumor, which may indicate locally advanced or metastatic disease on presentation. Liang et al. reported a 10% prevalence of tumor thrombus in pelvic sarcomas, with a median overall survival of 14 months [26]. However, the survival rate for pediatric sarcomas of any location with tumor thrombus remains unknown.

Therefore, the purpose of this study is to examine the survival rate for pediatric patients with tumor thrombus in sarcoma and evaluate other risk factors for mortality in this patient population.

## 2. Materials and Methods

### 2.1. Patient Selection

**Figure 1:**
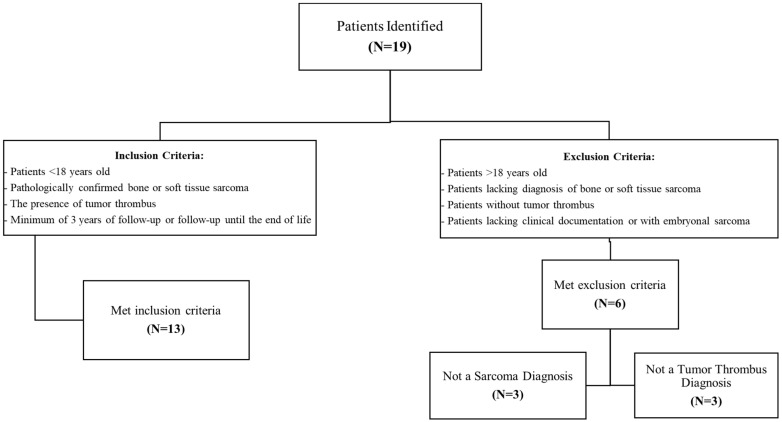
Inclusion and Exclusion Criteria.

A retrospective review was conducted using a pathology database from a pediatric tertiary care center between 1 January 1990, and 1 January 2025, for patients with tumor thrombus in sarcoma. Patients <18 years old were included if they had a pathologically confirmed bone or soft tissue sarcoma and the presence of tumor thrombus, with a minimum of 3 years of follow-up or follow-up until the end of life. Patients with tumor thrombus on initial staging studies or within 30 days of sarcoma diagnosis were considered to have thrombus at presentation. Patients lacking appropriate clinical documentation or with embryonal sarcomas were excluded. The initial review of the pathology database identified 19 patients, and after screening to confirm eligibility, 13 patients were included in the final analysis (Figure 1). This study received Institutional Review Board approval prior to beginning any study procedures.

### 2.2. Study Design

A retrospective chart review was conducted to gather patient demographics, clinical data, including surgical and medical management of the tumor, pathology reports, relevant imaging, and survival rates. Patient demographics included sex, age, tumor type, and tumor location. Dates of sarcoma diagnosis and tumor thrombus diagnosis were recorded. The sarcoma diagnosis was confirmed by reviewing the pathology reports of biopsied specimens. The tumor thrombus diagnosis was based on clinical notes and MRI imaging review by the senior authors. The date of death was determined through clinical notes. The follow-up duration was calculated from the date of sarcoma diagnosis until the last clinical follow-up note.

### 2.3. Statistical Analysis

Statistical analyses were conducted using STATA version 14.0 (StataCorp, College Station, TX, USA). Categorical variables were reported as counts and percentages. Continuous variables were presented as medians with the corresponding interquartile ranges (IQR). The primary outcome was survival rates. The secondary analysis involved examining the correlation between survival duration and clinical variables such as age, sex, and the presence of metastatic disease. A significance level of *p* < 0.05 was used for all analyses. All *p*-values reported are from Fisher’s exact tests or Wilcoxon rank sum tests.

## 3. Results

### 3.1. General Cohort Characteristics

Thirteen patients met the eligibility criteria and were included in the analysis. The cohort consisted of nine (69.2%) males and four (30.8%) females. The median age at the time of sarcoma diagnosis was 10.5 years (IQR 7.3, range: 3.4–17.0). Among those who underwent surgery, the median age at initial surgery was 11.6 years (IQR 7.1, range: 4.2–17.3). The median follow-up duration for surviving patients was 8.3 years (IQR 6.6, range: 3.1–11.5) (Table 1).

### 3.2. Tumor Characteristics and Initial Treatment

Nine patients were diagnosed with osteosarcoma, two with rhabdomyosarcoma, one with clear cell sarcoma, and one with desmoplastic small round cell tumor. Ten (76.9%) patients had a tumor confined to the bone, including three of the femur, three of the humerus, two of the pelvis, one of the sacrum, and one of the tibia. The remaining three (23.1%) patients had tumors restricted to soft tissues, including the abdomen, heart, and kidney. Twelve (92.3%) patients underwent surgery after neoadjuvant chemotherapy, and all of these patients resumed chemotherapy following the operation. The one patient who did not have surgery was treated with chemotherapy alone (Table 2).

Two (15.4%) patients had tumor thrombus at initial presentation during staging studies, while 11 (84.6%) patients were diagnosed with tumor thrombus at a later time. The median time from sarcoma diagnosis to tumor thrombus detection was 3.7 months (IQR 10.5, range: 1.3–126.2). Eight (61.5%) patients developed tumor thrombus locally at the sarcoma’s anatomic site, whereas five (38.5%) developed tumor thrombus at a location not contiguous with the primary tumor. Among the noncontiguous cases, three out of five developed tumor thrombus later after initial presentation, and four out of five had metastatic disease at presentation (Table 3).

### 3.3. Primary Analysis

**Figure 2:**
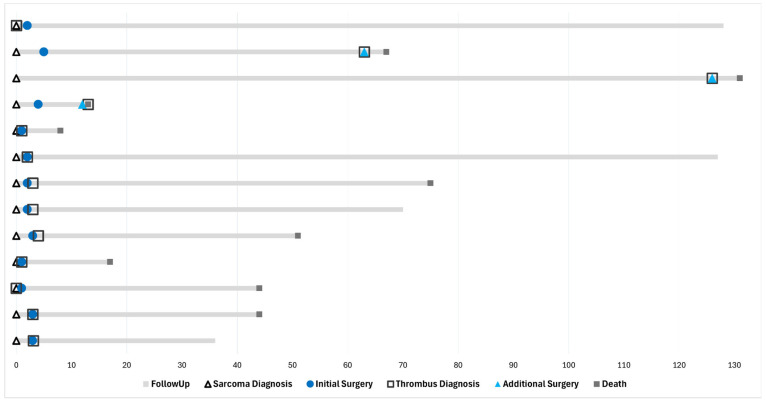
Swimmer Plot of Disease Course and Treatment.

Nine (69.2%) patients did not survive after tumor thrombus diagnosis, resulting in an overall survival rate of 30.8%. The median age at the time of death was 14.4 years (IQR 4.9, range: 4.8–17.8). The median duration from sarcoma diagnosis to death was 44.2 months (IQR 50.8, range: 8.2–131.6). The median time from index surgery to death was 41.4 months (IQR 42.7, range: 6.9–73.4). The median time from tumor thrombus diagnosis to death was 15.2 months (IQR 38.8, range: 0.5–71.9) (Table 4) (Figure 2).

### 3.4. Secondary Analysis

Seven (53.8%) patients had localized disease at the initial sarcoma diagnosis, while 6 (46.2%) had metastatic disease. Of those with metastatic disease, 5/6 (83.3%) did not survive, and 4/7 (57.1%) with localized disease did not survive. Metastatic disease was not found to be predictive of a shorter time from sarcoma diagnosis to death (*p* = 0.624), tumor thrombus diagnosis to death (*p* = 0.624), nor was it a significant predictor of death (*p* = 0.559) (Table 5). Additionally, noncontiguous tumors were not found to be predictive of a shorter time from sarcoma diagnosis to death (*p* = 0.086), from tumor thrombus diagnosis to death (*p* = 0.462), nor was it a significant predictor of death (*p* = 1.000). Age was not predictive of the time to death from tumor thrombus diagnosis (*p* = 0.119). Sex was also not predictive of the time to death from tumor thrombus diagnosis (*p* = 0.796).

## 4. Discussion

To our knowledge, this is the only reported cohort of pediatric patients with tumor thrombus in sarcoma and the first to demonstrate the survival rate in such patients. We observed a 31% survival rate at a median follow-up of 8 years, with death occurring a median of 15 months after tumor thrombus diagnosis. The low survival rate in these patients was found to be independent of patient demographics and the presence of metastatic disease.

Literature on tumor thrombus in sarcoma is limited. Yedururi et al. described 20 adult patients with pelvic osteosarcoma and tumor thrombus. Their study found a 5-year survival rate of 45% for patients who underwent surgery and 0% for those who did not, resulting in an overall survival rate of 25%. They also observed that the presence of tumor thrombus in surgically treated patients was linked to lower survival compared to those without tumor thrombus [12]. Our study similarly reported a low survival rate of 31%, but several key differences should be noted. Their research included only pelvic osteosarcomas, whereas the current study analyzed all sarcomas of soft tissue and bone. Additionally, they focused solely on localized tumor thrombus in large draining veins of the pelvis or abdomen, while our study described thrombus both locally and at noncontiguous sites to the primary tumor, which occurred in 38.5% of our patients. Finally, their study involved patients under 45 years old, whereas our research included only pediatric patients to offer insight into this unique group. A study by Liang et al., which excluded patients under 18, examined individuals with only osseous sarcomas of the pelvis and found a 10% prevalence of tumor thrombus with a median survival of 14 months [26]. Although patients in their cohort experienced a survival benefit from surgical intervention, tumor thrombus was consistently associated with a poor prognosis across nearly all outcome measures [26]. Our findings are similar, with a median time to death from thrombus diagnosis of 15 months. However, that study specifically included pelvic bone sarcomas in adults, whereas our research encompasses all sites of sarcoma within a pediatric-specific population.

The low survival rate in patients with tumor thrombus was independent of age, sex, and the presence of metastatic disease in our study, as none of these factors predicted mortality. Similar to existing literature in adults [26], there was no significant difference in survival rates between patients with localized disease and those with metastatic disease. However, there was a non-significant trend toward a shorter time to death after thrombus diagnosis in patients with metastatic disease. While it is known that patients with metastatic sarcoma have both higher mortality and shorter lifespans [27,28], our findings suggest that tumor thrombus itself is a predictor of poor prognosis, along with the presence of distant metastases. While our study is not adequately powered to describe the relationship between tumor thrombus and metastatic disease, the fact that tumor thrombus is a negative prognostic factor in either scenario is an important finding, especially given that this is a rare event in a rare group of diseases.

Tumor thrombus was found to be noncontiguous with the anatomic site of the sarcoma in 38.5% of patients, with the pulmonary veins being the most common site. Most of these patients had their thrombus discovered after initial presentation and also had metastatic disease elsewhere. Aside from single-patient case reports [21,22,23,24,25], the existing literature on tumor thrombus in sarcoma generally describes thrombus development adjacent to the primary tumor [12,26]. Our study highlights the potential for pediatric sarcoma patients to develop tumor thrombus in the venous system outside of the areas near the primary tumor, which indicates advanced, aggressive disease. Therefore, we recommend careful examination and a low threshold for advanced imaging to help diagnose tumor thrombus in patients where there is a high index of suspicion, such as those with unilateral limb swelling, edema, varicosities, or other signs of venous congestion, as these are unusual findings in pediatric patients.

This study has limitations. As is common in sarcoma research, our sample size is small due to the rarity of pediatric sarcoma and the presence of tumor thrombus in such cases. However, by including all patients over a 35-year period from one of the largest pediatric hospitals in the United States, we have presented the largest cohort of pediatric patients with tumor thrombus in sarcoma to date. Additionally, this was a retrospective study conducted at a single large tertiary care center, and with inherent selection bias, our findings may not be generalizable to the broader population. Finally, we acknowledge potential confounding factors related to survival rates in malignancy, such as the aggressiveness of the primary disease, the development of metastasis, and chemotherapy-induced medical frailty. Nonetheless, we controlled for the presence of metastatic sarcoma through our secondary analysis, which showed that tumor thrombus affects mortality independent of metastatic disease. Future directions should include a multicenter collaborative study to increase sample size and include a more diverse pediatric cohort.

## 5. Conclusions

In conclusion, the survival rate for pediatric sarcoma with tumor thrombus is low, with patients generally not surviving more than 15 months after diagnosis, and surgical intervention did not appear to result in long-term survival in this small series. Tumor thrombus in this population can be noncontiguous with the primary sarcoma location, requiring a low threshold for screening with advanced imaging. Clinicians should consider these findings when counseling patients, developing treatment plans, and assessing surgical eligibility.

## Figures and Tables

**Table 1:** Patient Demographics.

	N = 13
Age at Sarcoma Diagnosis (years)	10.5 (IQR 7.3, range: 3.4–17.0)
Age at Surgery (years)	11.6 (IQR 7.1, range: 4.2–17.3)
Length of Follow-up of Living Patients (years)	8.3 (IQR 6.6, range: 3.1–11.5)
Sex	
Male	9 (69.2%)
Female	4 (30.8%)

All categorical values are presented as *n* (%) and continuous values as median (interquartile range, range).

**Table 2:** Tumor Characteristics and Initial Treatment.

	N = 13
Tumor Type	
Osteosarcoma	9 (69.2%)
Rhabdomyosarcoma	2 (15.4%)
Clear Cell Sarcoma	1 (7.7%)
Small Round Cell Tumor	1 (7.7%)
Tumor Location	
Femur	3 (23.1%)
Humerus	3 (23.1%)
Pelvis	2 (15.4%)
Sacrum	1 (7.7%)
Tibia	1 (7.7%)
Abdomen	1 (7.7%)
Heart	1 (7.7%)
Kidney	1 (7.7%)
Treatment Type	
Neoadjuvant and/or Adjuvant Chemotherapy	13 (100%)
Surgical Intervention	12 (92.3%)

All values are presented as *n* (%).

**Table 3:** Patients with Tumor Thrombus Noncontiguous to Primary Tumor.

Patient Number	Sarcoma Location	Thrombus Location	Thrombus Developed Later	Metastatic at Initial Diagnosis
1	Pelvis	Femur	No	Yes
2	Tibia	Femur	Yes	Yes
3	Pelvis	Lung	Yes	No
4	Humerus	Lung	Yes	Yes
5	Femur	Lung	No	Yes

**Table 4:** Survival Rate.

	N = 13
Survival Rate	4 (30.8%)
Age at Time of Death (years)	14.4 (IQR 4.9, range: 4.8–17.8)
Time from Sarcoma Diagnosis to Death (months)	44.2 (IQR 50.8, range: 8.2–131.6)
Time from Index Surgery to Death (months)	41.4 (IQR 42.7, range: 6.9–73.4)
Time from Thrombus Diagnosis to Death (months)	15.2 (IQR 38.8, range: 0.5–71.9)

All categorical values are presented as *n* (%) and continuous values as median (interquartile range, range).

**Table 5:** Localized vs. Metastatic Disease and Survival Rate.

	Localized (N = 7)	Metastatic (N = 6)	*p*-Value
Survival Rate	4 (57.1%)	5 (83.3%)	0.559
Time from Sarcoma Diagnosis to Death (months)	47.7 (IQR 61.0, range: 17.0–131.6)	44.2 (IQR 54.1, range: 8.2–75.9)	0.624
Time from Thrombus Diagnosis to Death (months)	27.7 (IQR 33.1, range: 5.5–46.5)	6.9 (IQR 39.9, range: 0.5–71.9)	0.624
	Contiguous (N = 8)	Noncontiguous (N = 5)	
Time from Sarcoma Diagnosis to Death (months)	39.3 (IQR 34.4, range: 8.2–75.9)	56.0 (IQR 70.8, range: 13.7–131.6)	0.086
Time from Thrombus Diagnosis to Death (months)	40.3 (IQR 31.3, range: 6.9–71.9)	4.9 (IQR 22.4, range: 0.5–44.2)	0.462

All categorical values are presented as *n* (%) and continuous values as median (interquartile range, range).

## Data Availability

The data presented in this study are available on request from the corresponding author due to personal health information restrictions.

## References

[1] Burningham Z., Hashibe M., Spector L., Schiffman J.D. (2012). The epidemiology of sarcoma. Clin. Sarcoma Res..

[2] Skubitz K.M., D’Adamo D.R. (2007). Sarcoma. Mayo Clin. Proc..

[3] Popovich J.R., Kashyap S., Gasalberti D.P., Cassaro S. (2025). Sarcoma.

[4] Anderson J.L., Park A., Akiyama R., Tap W.D., Denny C.T., Federman N. (2015). Evaluation of In Vitro Activity of the Class I PI3K Inhibitor Buparlisib (BKM120) in Pediatric Bone and Soft Tissue Sarcomas. PLoS ONE.

[5] Ou J.Y., Spraker-Perlman H., Dietz A.C., Smits-Seemann R.R., Kaul S., Kirchhoff A.C. (2017). Conditional survival of pediatric, adolescent, and young adult soft tissue sarcoma and bone tumor patients. Cancer Epidemiol..

[6] McEvoy M.T., Siegel D.A., Dai S., Okcu M.F., Zobeck M., Venkatramani R., Lupo P.J. (2023). Pediatric rhabdomyosarcoma incidence and survival in the United States: An assessment of 5656 cases, 2001–2017. Cancer Med..

[7] Wells M.E., Eckhoff M.D., Davis W., Singh V., Rajani R., Polfer E.M. (2024). Ewing Sarcoma in the Pediatric Population: Predictors of Survival Within the United States. J. Am. Acad. Orthop. Surg. Glob. Res. Rev..

[8] Tathireddy H., Rice D., Martens K., Shivakumar S., Shatzel J. (2023). Breaking down tumor thrombus: Current strategies for medical management. Thromb. Res..

[9] Quencer K.B., Friedman T., Sheth R., Oklu R. (2017). Tumor thrombus: Incidence, imaging, prognosis and treatment. Cardiovasc. Diagn. Ther..

[10] Shang B., Guo L., Shen R., Cao C., Xie R., Jiang W., Wen L., Bi X., Shi H., Zheng S. (2021). Prognostic Significance of NLR About NETosis and Lymphocytes Perturbations in Localized Renal Cell Carcinoma with Tumor Thrombus. Front. Oncol..

[11] Marcoux C. (2019). Natural History of Tumor Thrombus: A Single-Centre Retrospective Study. Blood.

[12] Yedururi S., Chawla S., Amini B., Wei W., Salem U.I., Morani A.C., Wang W.L., Gorlick R., Lewis V.O., Daw N.C. (2018). Tumor thrombus in the large veins draining primary pelvic osteosarcoma on cross sectional imaging. Eur. J. Radiol..

[13] National Comprehensive Cancer Network NCCN Clinical Practice Guidelines in Oncology (NCCN Guidelines^®^): Soft Tissue Sarcoma (Version 1.2026). http://www.nccn.org/professionals/physician_gls/pdf/sarcoma.pdf.

[14] National Comprehensive Cancer Network NCCN Clinical Practice Guidelines in Oncology (NCCN Guidelines^®^): Bone Cancer (Version 2.2026). http://www.nccn.org/professionals/physician_gls/pdf/bone.pdf.

[15] Bertrand T.E., Cruz A., Binitie O., Cheong D., Letson G.D. (2016). Do Surgical Margins Affect Local Recurrence and Survival in Extremity, Nonmetastatic, High-grade Osteosarcoma?. Clin. Orthop..

[16] Potter B.K., Hwang P.F., Forsberg J.A., Hampton C.B., Graybill J.C., Peoples G.E., Stojadinovic A. (2013). Impact of margin status and local recurrence on soft-tissue sarcoma outcomes. J. Bone Jt. Surg. Am..

[17] Jang W.Y., Kim H.S., Han I. (2021). Impact of surgical margin on survival in extremity soft tissue sarcoma: A systematic review and meta-analysis. Medicine.

[18] Stevenson J.D., Laitinen M.K., Parry M.C., Sumathi V., Grimer R.J., Jeys L.M. (2018). The role of surgical margins in chondrosarcoma. Eur. J. Surg. Oncol. J. Eur. Soc. Surg. Oncol. Br. Assoc. Surg. Oncol..

[19] Poultsides G.A., Tran T.B., Zambrano E., Janson L., Mohler D.G., Mell M.W., Avedian R.S., Visser B.C., Lee J.T., Ganjoo K. (2015). Sarcoma Resection With and Without Vascular Reconstruction: A Matched Case-control Study. Ann. Surg..

[20] Davis L.A., Dandachli F., Turcotte R., Steinmetz O.K. (2017). Limb-sparing surgery with vascular reconstruction for malignant lower extremity soft tissue sarcoma. J. Vasc. Surg..

[21] Navalkele P., Jones S.M., Jones J.K., Salazar J.D., Toy P.C., Iyer R.V., Herrington B. (2013). Osteosarcoma tumor thrombus: A case report with a review of the literature. Tex. Heart Inst. J..

[22] Doya L.J., Alyousef K., Oukan M., Razzok A., Alshabab B.S., AlEid T., Saloum R., Nasser H. (2022). Clear cell sarcoma of the kidney with inferior vena cava thrombus: A case report. J. Med. Case Rep..

[23] Atagi K., Karashima T., Mizutani K., Fukuhara H., Fukata S., Miura Y., Mitsuishi A., Hanazaki K., Uemura S., Miyazaki R. (2023). Primary adrenal Ewing’s sarcoma family of tumors with tumor thrombus of the inferior vena cava: A case report. J. Med. Case Rep..

[24] Tripathy S., Shamim S.A., Chellapuram S., Barwad A., Rastogi S. (2020). Primary Ewing Sarcoma/Primitive Neuroectodermal Tumor of Kidney With Inferior Vena Cava Thrombus: Findings on 18F-FDG PET/CT. Clin. Nucl. Med..

[25] Nakata T., Kawano H., Hayashi T., Koga S., Ikeda S., Maemura K. (2021). Tumor Thrombus of Sacral Osteosarcoma Under Inferior Vena Cava Filter. Circ. J..

[26] Liang H., Guo W., Tang X., Yang R., Yan T., Yang Y., Ji T., Sun X., Xie L., Xu J. (2021). Venous Tumor Thrombus in Primary Bone Sarcomas in the Pelvis: A Clinical and Radiographic Study of 451 Cases. J. Bone Jt. Surg. Am..

[27] Carbonnaux M., Brahmi M., Schiffler C., Meeus P., Sunyach M.P., Bouhamama A., Karanian M., Tirode F., Pissaloux D., Vaz G. (2019). Very long-term survivors among patients with metastatic soft tissue sarcoma. Cancer Med..

[28] SEER*Explorer: An Interactive Website for SEER Cancer Statistics [Internet]. Surveillance Research Program, National Cancer Institute. https://seer.cancer.gov/explorer/.

